# Breaking the Silence: A Mixed Methods Analysis Unveiling Barriers to Mental Health Service Use in Breast Cancer Survivors With Clinically Significant Distress

**DOI:** 10.1002/pon.70599

**Published:** 2026-10-27

**Authors:** Ana Danner, Tayler Gowan, Madison Schwarz, Matthew Hays, Yang Li, Shelley A. Johns

**Affiliations:** ^1^ Indiana University School of Medicine Indianapolis Indiana USA; ^2^ Department of Biostatistics and Health Data Science Indiana University School of Medicine Indianapolis Indiana USA; ^3^ Richard M. Fairbanks School of Public Health Indiana University Indianapolis Indianapolis Indiana USA; ^4^ William M. Tierney Center for Health Services Research Regenstrief Institute, Inc. Indianapolis Indiana USA

**Keywords:** anxiety, avoidance behavior, breast neoplasms, cancer survivors, depression, fear, mental health services, mixed methods study, psychological distress

## Abstract

**Background:**

Breast cancer survivors are at elevated risk for psychological distress, including depression, anxiety, and post‐traumatic stress (PTS), compared to healthy controls. Fear of cancer recurrence (FCR) is particularly prevalent, with approximately 50% of breast cancer survivors reporting clinically significant FCR.

**Aims:**

This mixed methods study examined associations among psychological distress, mental health service (MHS) use, and barriers to MHS use among breast cancer survivors.

**Methods:**

Using a sequential explanatory mixed methods design, we analyzed baseline data from 384 early‐stage, post‐treatment breast cancer survivors with clinically significant FCR enrolled in a randomized controlled trial. Associations between distress symptoms and MHS use were examined. Subsequently, qualitative interviews were conducted with 24 distressed breast cancer survivors to explore barriers to MHS use.

**Results:**

Of 298 (77.81%) breast cancer survivors with at least one clinically significant distress measure, only 61 (20.47%) reported MHS use within the prior 3 months. Anxiety (*p* = 0.0030), depression (*p* = 0.0015), and PTS (*p* = 0.0387) symptoms were significantly associated with MHS use, whereas FCR was associated with fewer MHS visits. Interviews revealed barriers including avoidant coping, financial and logistical challenges, and limited access to timely, integrated care, as well as suggestions for improved support.

**Conclusions:**

Few clinically distressed breast cancer survivors utilize MHS. Anxiety, depression, and PTS symptoms may better predict MHS use than FCR, potentially due to avoidant coping among fearful survivors. Interventions targeting avoidance, alongside survivor‐informed improvements in mental health care delivery, may enhance MHS access and outcomes.

**Trial Registration:**

NCT05364450

## 1 Background

Globally, nearly 8 million women are living following a breast cancer diagnosis within the past 5 years, making them the largest cancer survivor population worldwide [1]. These survivors are at increased risk for psychological distress, including depression and anxiety, for at least 10 years post‐diagnosis [2]. Fear of cancer recurrence (FCR) is a prevalent form of distress and unmet need in cancer survivors, negatively affecting quality of life, medical follow‐up behavior, relationships, and mood [3]. Approximately 50% of breast cancer survivors report clinically significant FCR [4].

Although clinical guidelines recommend evidence‐based treatments for distress [5] and management of depression and anxiety [6] in adult cancer survivors, many cancer care centers lack readily available, adequately trained providers or sufficient infrastructure to implement them consistently [7]. Moreover, despite higher distress, survivors do not utilize mental health services (MHS) more than the general population, suggesting significant under‐utilization [8].

Both traditional cognitive behavioral therapy (CBT) and modern acceptance and commitment therapy (ACT) have demonstrated efficacy in addressing FCR [9, 10]. ACT proposes that psychological suffering worsens with experiential avoidance, whereas psychological flexibility supports engagement in values‐consistent action even in the presence of distress [11, 12]. FCR is often maintained through unhelpful cognitive and behavioral processes such as threat monitoring, reassurance seeking, avoidance, and intolerance of uncertainty [13, 14]. As such, ACT may be particularly well‐suited to address avoidant coping and promote values‐aligned behavior [15]. The efficacy of group‐based ACT versus CBT for FCR among breast cancer survivors is currently being evaluated in a large randomized controlled trial (RCT; NCT05364450). The present study is a secondary analysis of baseline survey data from trial participants, all of whom reported clinically significant FCR at eligibility screening. Objectives of this analysis included (1) to assess the prevalence and patterns of psychological distress in breast cancer survivors with clinically significant FCR and (2) to explore associations between distress and MHS use. Given the theoretical importance of psychological flexibility within ACT, we examined associations between its operationalized counterpart, psychological inflexibility, and FCR in this population. Limited MHS use among distressed breast cancer survivors prompted follow‐up qualitative focus groups to explore perceptions of MHS, barriers to access and utilization, and survivor‐informed recommendations for improving support.

## 2 Methods

### 2.1 Study Design

A two‐phase, mixed methods, sequential explanatory design was used. The initial quantitative phase involved a secondary analysis of baseline data from 384 early‐stage breast cancer survivors enrolled in a three‐arm RCT investigating the efficacy of an ACT intervention in breast cancer survivors with clinically significant FCR reported at eligibility screening. Participants were recruited from cancer centers affiliated with the Indiana University (IU) Simon Comprehensive Cancer Center between August 2021 and July 2023. All study procedures were approved by the IU Institutional Review Board, and informed consent was obtained from all participants. Participants completed self‐report questionnaires via the secure Research Electronic Data Capture (REDCap) web application at baseline and received a $40 gift card for survey completion. The present analysis examined baseline responses to assess the prevalence of psychological distress—including FCR, anxiety, depression, and post‐traumatic stress (PTS)—and their associations with MHS use.

Following the quantitative phase, a qualitative follow‐up was conducted to better understand participants' perceptions of MHS and identify barriers to care.

### 2.2 Study Population

The quantitative sample consisted of 384 female breast cancer survivors who met all inclusion criteria and none of the exclusion criteria in Table 1.

**TABLE 1:** Summary of participant eligibility criteria.

Inclusion criteria	Exclusion criteria
Age ≥ 18 years Diagnosis of stage I‐IIIA breast cancer without evidence of distant disease at time of study entry Completion of primary treatment (surgery, chemotherapy, radiation therapy, and/or targeted treatment) within the past 5 years (ongoing endocrine therapy was allowed) Clinically significant fear of cancer recurrence (FCR), defined as a score ≥ 17 on the 7‐item FCR screening tool (FCR7) [16] at screening Ability to speak and read English	Previous cancer diagnosis besides breast (non‐melanoma skin cancer or melanoma in situ was allowed) Concurrent participation in another research study that has the potential to skew results of this study or the other study in which the person was participating Comorbidities, medications, or deficits that would impair participation in the trial, including: History of stroke, encephalitis, traumatic brain injury/surgery, Alzheimer's disease, or other dementia Severe depressive symptoms (PHQ‐2 score ≥ 5 at screening) [17] Active substance use disorder or uncontrolled bipolar disorder, psychosis, or schizophrenia Obvious hearing and/or communicative disability Opt‐out of pre‐screening for research studies noted in the electronic medical record

Abbreviation: PHQ‐2, Patient Health Questionnaire‐2.

### 2.3 Measures

*Fear of Cancer Recurrence* was assessed using the FCR7, which assesses worry, anxiety, and strong emotional responses related to cancer recurrence, as well as cognitive and behavioral reactions; scores of ≥ 17 are considered clinically significant [16].

*Anxiety* was assessed using the Generalized Anxiety Disorder scale (GAD‐7); scores ≥ 10 indicate clinically significant anxiety [18].

*Depressive Symptoms* were assessed using the 8‐item Patient Health Questionnaire depression scale (PHQ‐8), which assesses how often participants have experienced depressive symptoms in the past 2 weeks; scores ≥ 10 are considered clinically significant [19].

*Post‐Traumatic Stress* was assessed using the 22‐item Impact of Events Scale‐Revised (IES‐R), which measures symptoms of intrusion, avoidance, and hyperarousal; scores ≥ 33 indicate clinically significant PTS symptoms [20].

*Mental Health Service Use* was assessed using four items (Supporting Information S1: Table 1A) from the Treatment Inventory of Costs in Patients with Psychiatric Disorders (TiC‐P), including outpatient, private practice, and hospital‐based MHS and support groups [21]. Participants reported how many times they used each service in the 3 months prior to baseline.

*Psychological Inflexibility* was assessed using the 18‐item Cancer Acceptance and Action Questionnaire (CAAQ), which measures psychological inflexibility—also known as experiential avoidance—regarding cancer‐related thoughts and emotions [22]. Mean scores range from 1 to 7; higher scores indicate greater psychological inflexibility and, consequently, a higher tendency toward avoidant coping [23].

### 2.4 Statistical Analyses

Participant demographics, cancer diagnoses, and prior cancer treatments were described by summary statistics. Continuous variables were described using mean [standard deviation (SD)], and categorical variables were summarized by frequencies (relative frequencies in percentage). FCR7, GAD‐7, PHQ‐8, and IES‐R sums were each calculated on subjects who had complete‐item responses on the measure. Distress measures were compared between the FCR7 severity groups based on the 7‐item FCR scale (FCR7) by the Kruskal‐Wallis test. Associations between clinical significance of psychological distress scores and any usage of MHS were assessed using the Chi‐square or Fisher's Exact tests. Poisson regression was employed to further evaluate the association between psychological distress scores and log‐count of MHS visits. The relationship between FCR and psychological inflexibility was measured by the Spearman's correlation coefficient (*ρ*) to indicate the strength and direction of the correlation. *p‐*values below 0.05 were defined as statistically significant. No formal adjustments were made for multiple testing because analyses were exploratory; this approach prioritizes the detection of potential signals (minimizing Type II error) over strict control of false positives [24].

### 2.5 Qualitative Design

Participants eligible for the qualitative phase were a purposive subset of the RCT sample who had consistently reported two or more clinically significant distress scores (FCR, anxiety, depression, and/or PTS) and no MHS use across the one‐year study period. Eligible participants for this portion of the study had also opted in to be contacted regarding study opportunities beyond their completion of the parent RCT. This sampling approach was intended to capture perspectives from breast cancer survivors experiencing persistent distress without engagement in MHS, rather than to achieve sample representativeness. Participants were recruited from this eligible pool based on availability and willingness to participate, and recruitment continued until data saturation was achieved. In total, 24 eligible and consenting participants completed a single virtual focus group interview via Zoom Health. Eight total focus group interviews were conducted with an average of three survivors each. Focus groups were led by the same bachelors‐trained qualitative interviewer using a semi‐structured guide containing five open‐ended questions designed to explore participants' perceptions of MHS, factors influencing their motivation to seek services, and suggestions for improving breast cancer survivors' psychosocial care (Supporting Information S1: Table 2A). Interviews ranged between 58 and 90 minutes. Each interviewee received a $100 gift card for their participation.

All focus groups were audio‐ and video‐recorded and transcribed verbatim. At least two members of the qualitative analytic team independently reviewed each transcript and identified salient quotes and preliminary themes related to barriers to MHS use and suggestions for improvement. Initial findings were discussed during regular team meetings to develop a shared codebook. Discrepancies in coding or theme identification were resolved through iterative discussion and consensus. Final coding was conducted independently by multiple team members and then compared to ensure consistency and reliability. NVivo qualitative data analysis software was used for data organization and coding. Coded data were then grouped into conceptual themes (Tables 4 and 5).

## 3 Results

### 3.1 Quantitative Phase

#### 3.1.1 Demographics

Demographic data and clinical characteristics of the quantitative sample are detailed in Table 2. To contextualize the data, the study sample was compared with the IU Simon Comprehensive Cancer Center catchment area (i.e., the state of Indiana). The mean age of participants was 56.3 years (SD = 11.2), consistent with a state population in which 17.5% are aged ≥ 65 years (vs. 18.0% nationally). The sample was predominantly non‐Hispanic white (81.8%), slightly higher than the state proportion (75.2%; 57.5% nationally), with Black or African American participants comprising 14.0% (vs. 10.6% statewide). Educational attainment was higher in the study sample, with 71.4% holding a college degree compared to 30.7% of Indiana residents with a bachelor's degree or higher [25].

**TABLE 2:** Participant demographics and clinical characteristics.

	*N* = 384
Age, mean (SD)	56.3 (11.2)
Race (*n* = 378), *n* (%)	
White	309 (81.75%)
Black or African American	53 (14.02%)
Other	16 (4.23%)
Ethnicity (*n* = 368), *n* (%)	
Hispanic or Latino/*a*	6 (1.63%)
Not hispanic or Latino/*a*	362 (98.37%)
Partner status (*n* = 383), *n* (%)	
Partnered	262 (68.41%)
Not partnered	121 (31.59%)
College degree, *n* (%)	274 (71.35%)
Employment, *n* (%)	
Employed full‐time	185 (48.18%)
Employed part‐time	29 (7.55%)
Retired	88 (22.92%)
Unable to work due to health/disability	30 (7.81%)
Other	52 (13.54%)
Income situation (*n* = 381), *n* (%)	
Comfortable	254 (66.67%)
Just enough to make ends meet	100 (26.25%)
Not enough to make ends meet	27 (7.09%)
Months since cancer diagnosed (*n* = 369), mean (SD)	35.98 (66.35)
Cancer Treatment(s) received, *n* (%)	
Surgery	358 (93.23%)
Radiation therapy	292 (76.04%)
Chemotherapy	206 (53.65%)
Hormone/endocrine therapy	268 (69.79%)
Targeted therapy	33 (8.59%)
Other	12 (3.13%)
Cancer stage, *n* (%)^a^	
IA/IB (stage 1)	234 (60.16%)
IIA/IIB (stage 2)	123 (32.03%)
IIIA (stage 3)	30 (7.81%)
FCR7 sum (*n* = 375), mean (SD)	21.6 (6.9)
GAD‐7 sum (*n* = 377), mean (SD)	5.8 (5.0)
PHQ‐8 sum (*n* = 373), mean (SD)	6.1 (5.0)
IES‐R sum (*n* = 351), mean (SD)	24.0 (14.1)

Abbreviations: N, total sample size; *n*, frequency count or variable‐specific sample size if less than N; SD, standard deviation.

^a^ Study inclusion criteria limited participants to Stage I–IIIA breast cancer; therefore, Stage 0, Stage IIIB/IIIC, and Stage IV categories were not represented and are omitted from the table.

#### 3.1.2 Psychological Distress

At baseline, 280 participants (74.67%) maintained clinically significant FCR (Supporting Information S1: Table 3A). Beyond FCR, 59.16% (*n* = 226) reported at least one additional form of psychological distress (Supporting Information S1: Table 4A), including anxiety (22.28%), depressive symptoms (23.32%), and PTS (25.64%; Supporting Information S1: Tables 5–7 A). FCR severity was directly correlated with statistically significant escalations in all three distress measures (Supporting Information S1: Table 8A).

#### 3.1.3 Mental Health Service Use

Of the 298 (77.81%) survivors who reported at least one clinically significant distress score (including FCR) at baseline (Supporting Information S1: Table 9A), only 61 (20.47%) used any MHS in the preceding 3 months (Supporting Information S1: Table 10A). While clinically significant anxiety (*p* = 0.0030), depression (*p* = 0.0015), and PTS symptoms (*p* = 0.0387) were significantly associated with a higher probability of using any of the four MHS assessed, categorical clinically significant FCR was not (Supporting Information S1: Table 11A). However, continuous analysis revealed that a one‐point increase in FCR7 was associated with a 3% increase in outpatient MHS visits (exp(β) = 1.03, *p* = 0.0117) but a 5% decrease in the number of support group visits (exp(β) = 0.95, *p* < 0.0001; Supporting Information S1: Table 12A).

#### 3.1.4 Components of Acceptance and Commitment Therapy

FCR was strongly correlated with greater cancer‐related psychological inflexibility, and thus increased experiential avoidance (*ρ* = 0.631, *p* < 0.001), consistent with theoretical expectations of the ACT model (Figure 1A).

### 3.2 Qualitative Follow‐Up Phase to Contextualize Quantitative Findings

Qualitative data were used to explore survivors' perceptions of MHS and barriers contributing to low utilization.

#### 3.2.1 Demographics

Demographic data and clinical characteristics of the participants in the qualitative follow‐up phase are detailed in Table 3.

**TABLE 3:** Demographics and clinical characteristics for focus group participants.

	*N* = 24
Age, mean (SD)	52.92 (10.81)
Race, *n* (%)	
White	18 (75.00)
Black or African American	6 (25.00)
Ethnicity, *n* (%)	
Hispanic or Latino/*a*	1 (4.17)
Not hispanic or Latino/*a*	23 (95.83)
Partner status, *n* (%)	
Partnered	13 (54.17)
Not partnered	11 (45.83)
College degree, *n* (%)	17 (70.83)
Employment, *n* (%)	
Employed full‐time	15 (62.50)
Employed part‐time	1 (4.17)
Retired	3 (12.50)
Unable to work due to health/disability	2 (8.33)
Other	3 (12.50)
Income situation	
Comfortable	12 (50.00)
Just enough to make ends meet	9 (37.50)
Not enough to make ends meet	3 (12.50)
Months since cancer diagnosed, mean (SD)	34.59 (18.12)
Cancer Treatment(s) received, *n* (%)	
Surgery	22 (91.67)
Radiation therapy	17 (70.83)
Chemotherapy	12 (50.00)
Hormone/endocrine therapy	17 (70.83)
Targeted therapy	1 (4.17)
Other	2 (8.33)
Cancer stage, *n* (%)^a^	
IA/IB (stage 1)	11 (45.83%)
IIA/IIB (stage 2)	12 (50.00%)
IIIA (stage 3)	1 (4.17%)
FCR7 sum *n* = 24, mean (SD)	28.5 (4.4)
GAD‐7 sum (*n* = 23), mean (SD)	13.3 (4.6)
PHQ‐8 sum (complete *n* = 23), mean (SD)	12.6 (5.4)
IES‐R sum (complete *n* = 22), mean (SD)	40.0 (10.4)

Abbreviations: N, total sample size in the qualitative sample; *n*, frequency count or variable‐specific sample size in the qualitative sample if less than N; SD, standard deviation.

^a^ Study inclusion criteria limited participants to Stage I–IIIA breast cancer; therefore, Stage 0, Stage IIIB/IIIC, and Stage IV categories were not represented and are omitted from the table.

#### 3.2.2 Barriers to Accessing or Pursuing Mental Health Services

Survivor‐informed barriers to MHS were identified and organized into major themes and subthemes (Table 4). These barriers spanned multiple levels, including personal, interpersonal, and systemic, which often intersected in complex ways. Many survivors described internal conflicts about seeking help, while others faced significant logistical and structural challenges. These barriers were compounded by the context of cancer care and societal stigma, resulting in a complex web of obstacles to support.

**TABLE 4:** Summary of commonly reported barriers to mental health service use in breast cancer survivors.

Theme	Subthemes
Internal hesitancy	– Avoidant coping – Feeling undeserving or not qualifying – Desire or obligation to cope alone – Discomfort and uncertainty surrounding MHS utility and outcomes – Lack of awareness of one's emotional needs
Therapy‐specific barriers	– Previous negative experience with MHS – Therapeutic burnout – Difficulty finding a compatible provider – Desire for a provider with shared backgrounds or lived experiences – Language barriers
Cost and insurance barriers	– High out‐of‐pocket costs – Challenges navigating insurance coverage
Competing responsibilities	– Prioritizing care for others – Time constraints – Work‐related barriers
Accessibility, availability, and convenience barriers	– Long wait times or practices not accepting new patients – Inconvenient appointment times – Lack of response from therapist or organization – Preference for in‐person (vs. virtual) care – Difficulty knowing where or how to start finding care
Cancer‐specific barriers	– Appointment fatigue from other medical care – Focused on survival during treatment—no capacity for MHS at the time offered
Societal influence and stigma	– Feeling forgotten or dismissed by society – External pressure to appear “okay” or “move on” – Perceived judgment from others

Abbreviation: MHS, mental health services.

##### 3.2.2.1 Internal Hesitancy

*Internal hesitancy* emerged as a prominent barrier. Many survivors described a tendency toward *avoidant coping*, fearing that engaging with emotions might intensify distress. As the participant with Study Identification Number (SID) 255 explained, “I just don't want to feel…It's like opening the floodgates. I'm afraid when those floodgates open, it's gonna be really not pleasant.” SID 291 noted their preference for emotional distancing: “I like to live in the land of denial.” Even when aware of their distress, some *questioned whether they deserved care or qualified for services:* “I feel like it would be complaining, because people have been in a worse situation than I have” (SID 374). Others worried about using resources meant for more severe cases: “I felt like, ‘Oh, this is a resource for stage IV [cancer survivors]’…I should not capitalize on this limited resource” (SID 326).

Closely tied to these feelings was a *desire—or sense of obligation—to cope alone*, often shaped by upbringing or caregiving roles. “You didn't stop to complain. You just keep on going. And that's what I've always done,” shared SID 255. SID 021 described herself as “a ‘manage‐everything‐myself’ kind of person.” For many, *discomfort and uncertainty surrounding MHS* added to their hesitation. Participants voiced anxieties about emotional exposure, unclear expectations, and outcomes. SID 335 said, “One of my biggest anxieties is I'm going to break down and cry in front of this person…Why spend a couple hundred dollars to sob in front of someone when I could do that in my bathroom for free?” Others worried about therapy's long‐term scope: “Where does it end? Is it gonna spiderweb out? Do I go forever? Where do I feel the success?” (SID 107). Some were simply *unaware of their emotional needs* during treatment, overwhelmed by cancer care: “I was so encompassed with the disease. I don't know that I even thought about reaching out” (SID 021).

##### 3.2.2.2 Therapy‐Specific Barriers

Beyond personal hesitancy, survivors encountered *therapy‐specific barriers. Negative past experiences* often left lasting skepticism. SID 291 recalled that a therapist had “fallen asleep” during a session, which left them feeling dismissed and invalidated: “I thought, ‘I guess my problems aren't that bad.’” SID 003 remembered thinking to herself, “Maybe I just need to learn how to deal with things on my own,” after a therapist cried while listening to their story. Repeated attempts led some to *therapeutic burnout*, characterized by emotional exhaustion and diminished motivation to re‐engage in counseling, where restarting felt overwhelming: “I don't want to keep changing people. It's hard to go back and start from the beginning again” (SID 211). Others *struggled to find a compatible provider*, noting the emotional toll of trial and error: “If you aren't comfortable with your therapist, you're not going to be able to open up” (SID 255).

Several participants sought therapists with *specific lived experiences*, such as being a fellow survivor or sharing their racial or cultural background: “I wanted to find a woman who was Black who dealt with breast cancer patients…don't know if she exists” (SID 122). For these individuals, perceived shared experience was described as a way to facilitate comfort and understanding. *Language barriers* also limited emotional expression for some: “It's hard to share your feelings in a different language” (SID 211).

##### 3.2.2.3 Cost and Insurance Barriers

*Cost and insurance barriers* were widespread. Survivors described how *high out‐of‐pocket costs* deterred them from pursuing care, especially when coupled with doubts about therapy's usefulness: “It's almost like running through that cost‐benefit analysis in your head and automatically thinking, ‘the cost is too much’” (SID 335). *Insurance navigation* was described as burdensome and confusing, with coverage changes frequently disrupting care: “Our health plan changed, and the therapist was no longer included. That is just a nightmare” (SID 282). SID 326 cited this as the main reason for delaying MHS: “I don't see providers that aren't in network…not even knowing who is in network and what would be covered is certainly a [barrier]. I don't have time to research all that, I'm just gonna put a pin in it.” Others emphasized the emotional toll of insurance gatekeeping, with SID 343 stating, “I hate that insurance dictates our care.”

##### 3.2.2.4 Competing Responsibilities

For many survivors, *competing responsibilities*, such as caregiving roles and demanding jobs, left little time or energy to seek MHS. Participants frequently *prioritized others' care over their own:* “I would always prioritize one of my kid's appointments, but my own would fall by the wayside” (SID 326). Another reflected, “I'm a mom, I'm a nurse…I'm really good at taking care of other people, but not good about taking care of myself” (SID 343). Others described *time constraints* and mental overload from juggling appointments and work. SID 335 stated, “I can't live my whole life going from appointment to appointment” (SID 335). Many also commented on the mental strain of personal work required outside MHS: “Sometimes it's not just ‘go for an hour a week’…the emotional impact it has on you kind of digs into your free time” (SID 218). Finally, *work‐related barriers*, such as inflexible hours, limited paid time off (PTO), or guilt taking time off, were commonly cited: “We run out of PTO time. I don't think I'm a burden to [my employer]. But it still feels like it because I have to schedule time off.” (SID 095).

##### 3.2.2.5 Accessibility, Availability, and Convenience Barriers

Survivors noted significant barriers related to *accessibility, availability, and convenience*, including *long waitlists* and poor provider communication. Many expressed frustration with limited availability and delays: “You get on a wait list, and it takes *months*” (SID 109). Such wait times felt especially discouraging when distress was already severe: “When you are reaching for help, you are really needing it as soon as possible” (SID 211). *Inconvenient appointment times,* especially during work hours, further limited access; as one explained, “The times of the support groups were during when I was working, so I wasn't able to attend any of them” (SID 255). Others were disheartened by *lack of response* after initial outreach: “I did reach out to a therapist, and they just never responded” (SID 095). Though often viewed as a convenient alternative, some found virtual care unengaging and *preferred in‐person visits*: “Virtual is just too easy for me not to participate” (SID 374). Survivors also described *uncertainty about where or how to begin finding care*, particularly when MHS were not integrated into oncology care: “Where do you even start from? That's one of my barriers” (SID 257).

##### 3.2.2.6 Cancer‐Specific Barriers and Societal Influence and Stigma

Survivors also described cancer‐specific barriers and societal influences that shaped their use and perceptions of MHS. Many experienced *appointment fatigue*, feeling logistically unable to add therapy to an already overwhelming medical schedule: “If I have to go to one more doctor appointment, I'm gonna scream” (SID 282). Others described a *survival‐focused mindset* during treatment that left little space for emotional processing: “I was just focused on survival for that first year and a half” (SID 095). After treatment, participants often felt *forgotten or dismissed*, with ongoing emotional struggles overlooked: “Everyone's there for you when you're going through it, and then the world thinks you're better” (SID 066). *External pressure to appear “okay” or to “move on”* also played a role. As SID 122 noted, “Some people think, ‘You're done [with treatment]. You're fixed.’ But you're not.” Others described reluctance to seek MHS due to *fear of judgment or appearing weak*: “You feel like you have to be tough. I don't want to give up the illusion that I'm not as tough as I pretend” (SID 326). In some social or family circles, MHS remained taboo, further discouraging care: “[Having pursued MHS in the past] I always got the impression that since I was in therapy, it meant that I was ‘the messed up one’” (SID 218). These intersecting cancer‐specific and societal challenges created a complex set of barriers that hindered access to needed support.

#### 3.2.3 Survivor‐Informed Suggestions for Improved Support During and After Cancer Treatment

In addition to sharing barriers to accessing and pursuing MHS, breast cancer survivors also shared a range of actionable suggestions summarized in Table 5 to improve the accessibility, delivery, and integration of MHS into their care throughout and post‐treatment. These suggestions reflect a desire for more proactive, coordinated, and personalized support across both medical and community settings. Survivors emphasized the need for system‐level changes that acknowledge the long‐term emotional impact of cancer and the ongoing challenges of survivorship.

**TABLE 5:** Summary of suggestions for improved support throughout breast cancer treatment and survivorship.

Domain: Barrier themes addressed	Survivor‐informed suggestion	Selected representative quote
Access and equity: Cost and insurance barriers Accessibility, availability, and convenience barriers Societal influence and stigma	Expand or bundle insurance coverage	“…The concept of having it [MHS] kind of integrated in conjunction with your physical treatment would have been a lot more enticing to me…I guess that would be my perfect world answer…it's [MHS] not an additional cost necessarily, or if it was, it was rolled into the treatment cost. And then that it could be an actual part of the treatment.” (SID 335)
	Increase funding to increase accessibility	“Mental health providers need to be better reimbursed. They need more funding.” (SID 109)
	Offer appointments outside of business hours	“I found a counselor who sees me one evening a week so I don't have to worry about getting off work…I know again, that's a barrier, finding those providers, who can and are willing to work the off hours, but it sure helps a lot…If I had to leave work an hour every week, then I couldn't do it.” (SID 093)
	Promote virtual options	“I just think it's great to kind of know all I have to give is exactly what time and how long…there's no prior driving there.” (SID 107)
	Expand support in rural areas/empower patients and survivors to build peer‐led initiatives	“Even if somebody were to start…just a woman's support group…breast cancer survivors, one night a week at one of the coffee shops…Maybe there's not a therapist there and maybe it's not a professional sitting there. But we're all there to help each other, and to talk about it, and talk about what each one of us are going through. That's at least a start, right? And especially in these smaller communities, where there is nothing else, that's a place to start.”
	Support diversity of MHS providers	“I think in my case, [the biggest barrier to accessing MHS] is the fact that I couldn't find someone that speaks my language through my insurance here in the U.S.” (SID 211)
System integration: Cancer‐specific barriers Competing responsibilities Accessibility, availability, and convenience barriers	Integrate and offer MHS throughout treatment	“I think that would be so helpful just to automatically have [MHS] set up like with your navigation. Part of your package. That probably would have helped me a lot…I didn't think that I needed those services. But had they been offered and scheduled, I'm a person that will go to my appointment.” (SID 210)
	Coordinate appointment times to limit repetitive trips	“It would've been great to have the counseling available while I was there for chemo or radiation, so I didn't have to come back a different day just for that.” (SID 164)
	Offer holistic survivorship care—Diet, exercise, mental health, education	“At the end of treatment, I felt like I needed help with everything—how to eat better, how to mentally process it, how to get physically stronger. There should be something that wraps that all together.” (SID 093)
	Address “drop‐off” in care after end of treatment	“When we finished the treatment, you kind of feel lost. Okay, what can I do from now to help myself just emotionally?” (SID 211)
Navigation and resource awareness: Therapy‐specific barriers Internal hesitancy Cancer‐specific barriers Societal influence and stigma	Explain who and what MHS are for	“They should explain that mental health is for anyone going through cancer—not just if you're falling apart. I didn't know it was something people like me could use.” (SID 091)
	Provide referrals to/Directory of therapists who specialize in care for breast cancer survivors	“I don't know if [hospital system] could like give a list of providers or therapists they partner with…a way to at least streamline your own research so it's not just one more daunting task when you don't really know how to look for somebody…someone who specializes in breast cancer…I'm more likely to pick up the ball at that point rather than start the whole process all by myself.” (SID 218)
	Create a centralized hub for quickly accessing resources	“I wish there was just one place to go—a website or number—to find support groups, therapists, everything. It's too scattered. You don't have the energy to search all over.” (SID 218)
Cultural acceptance and emotional readiness: Internal hesitancy Societal influence and stigma Cancer‐specific barriers	Normalize the use of MHS among breast cancer survivors	“Maybe give some testimonials or be able to have some recordings of just those people [fellow breast cancer survivors using MHS] saying, ‘Hey, you know, you may not think this is for you, but we're just a group,’ or maybe just people explaining what it is would help with some of those questions [surrounding MHS].” (SID 343)
	Be proactive in bringing up FCR	“Nobody brought up fear of recurrence with me… And that was one of my biggest struggles. I wish they had just said, ‘This is normal, and here's what we can do about it.’” (SID 282)
	Offer or encourage peer‐to‐peer support	“We need AA for breast cancer.” (SID 066)

Abbreviations: FCR, fear of cancer recurrence; MHS, mental health services; SID, Study Identification Number.

## 4 Discusssion

### 4.1 Clinical Implications of Quantitative Findings

The quantitative findings of this study highlight a striking discrepancy between high rates of clinically significant psychological distress and low MHS use among breast cancer survivors. Consistent with prior literature, clinically significant anxiety, depression, and PTS symptoms were common in this sample [2, 26, 27]. Rates were similar to or modestly higher than prior estimates, likely reflecting the study's focus on survivors with clinically significant FCR [3]. Although FCR was the most prevalent distress type, it was not associated with greater MHS use; rather, greater FCR severity was associated with reduced use of certain services. Contrastingly, anxiety, depression, and PTS symptoms were more likely to prompt MHS engagement. This aligns with prior research suggesting FCR is often normalized or overlooked by survivors and providers, making it less likely to be seen as warranting intervention compared to other conditions [3, 28]. Some may perceive FCR as an unavoidable part of survivorship rather than a treatable concern.

Avoidant coping may help explain this pattern. Survivors with high FCR may suppress distress or disengage emotionally as self‐protection. In this study, FCR was strongly linked to greater psychological inflexibility, indicating a tendency toward avoidant coping. These behaviors may contribute to an inverse association between FCR severity and use of MHS. Although avoidant coping may offer short‐term relief, it may ultimately reinforce distress and deter help‐seeking, leading to worse outcomes [29, 30]. Given avoidance's role in worsening FCR, ACT‐based interventions targeting psychological inflexibility can reduce FCR by promoting acceptance and values‐based living [31, 32, 33]. For survivors fearing overwhelming therapy, ACT provides tools to handle difficult emotions, simultaneously reducing distress and increasing engagement with psychosocial survivorship care.

Ultimately, the fact that only 20.47% of participants with clinically significant distress accessed any MHS highlights limitations in current survivorship care. These findings suggest that survivorship care may be enhanced by proactive screening, normalized discussion of psychosocial support, and accessible referral options, while respecting survivors' preferences, including the choice to manage distress independently. More broadly, the goal is to offer culturally responsive support without stigma so survivors can decide whether, when, and how to engage.

### 4.2 Clinical Implications of Qualitative Findings

The qualitative phase offers critical context for the quantitative trends, unveiling multifaceted barriers to MHS use, and underscoring the need for patient‐informed solutions. The discrepancy between high distress and low service utilization is not merely an issue of access, but of readiness, perception, and trust. Rooted in avoidant coping, self‐reliance, and stigma, survivors described internal hesitancy as a core obstacle to seeking care. These findings reinforce the importance of framing mental health care as a normal, expected part of survivorship as opposed to a last resort for those in crisis. Structural barriers such as cost, scheduling conflicts, long wait times, and difficulty finding a provider perceived as a good personal fit further reduced access. These issues align with international reports identifying similar challenges across oncology populations [34]. Survivors reported that logistical hurdles and their accompanying frustration may not only delay care‐seeking, but discourage it altogether.

While some prefer culturally concordant providers, such findings more broadly highlight the importance of fostering trust, rapport, and culturally responsive care across diverse patient–provider relationships, rather than reliance on demographic matching alone. This underscores the dual need for patient education to reassure survivors that effective care can be delivered across differences, alongside continued provider training in culturally responsive care to ensure all patients feel understood. Importantly, survivors suggested improving care via integrated MHS, flexible scheduling, telehealth, and routine FCR follow‐up. Liu et al. (2021) confirm the feasibility of similar structured educational interventions for FCR [35]. Furthermore, survivors highlighted the critical need for peer‐led and culturally responsive care models, aligning with recent calls for community‐embedded approaches [36, 37, 38].

These insights support the development of proactive, flexible, and survivor‐informed care models. Evidence‐based interventions, particularly if integrated early in survivorship and adapted to be trauma‐informed and culturally sensitive, may reduce psychological inflexibility and promote engagement with MHS.

### 4.3 Study Limitations

Several limitations should be noted. First, the data are based on self‐report measures, which may be subject to recall bias or social desirability effects. Second, both the quantitative and qualitative samples primarily comprised early‐stage, college‐educated, and predominantly white breast cancer survivors recruited from an academic medical center, which may limit generalizability to more diverse or medically underserved populations. Third, statistical analyses were conducted without adjustment for potential confounding variables, such as age and socioeconomic status. Finally, while the mixed methods design strengthens the interpretation of findings, the cross‐sectional nature of the quantitative phase precludes causal inference regarding relationships between psychological distress and MHS use. Longitudinal research is needed to assess how distress and care‐seeking behaviors evolve across the treatment and survivorship continuum.

## 5 Conclusion

This mixed methods study reveals a stark disconnect between the high prevalence of psychological distress among breast cancer survivors and their underuse of MHS. FCR, experienced by nearly three‐quarters of survivors at baseline, was not associated with increased MHS use and may even deter help‐seeking due to its strong link with avoidant coping.

To bridge this gap, future research and clinical practice must shift from passive, referral‐based screening toward integrated, proactive pathways embedded within oncology and survivorship care. Survivors' insights emphasize that future interventions must be flexible, accessible, and adaptable to the emotional, logistical, and cultural realities of life after cancer. Crucially, future studies must prioritize longitudinal designs and inclusive sampling across racially, culturally, and socioeconomically diverse backgrounds to clarify temporal relationships between distress and care‐seeking and to design equitable care models. Normalizing distress and implementing these survivor‐informed, evidence‐based approaches is vital to improving mental health engagement and emotional well‐being across the cancer continuum.

## Author Contributions

**Ana Danner:** conceptualization, data curation, formal analysis, investigation, methodology, project administration, visualization, writing – original draft, writing – review and editing. **Tayler Gowan:** formal analysis, methodology, project administration, resources, writing – original draft, writing – review and editing. **Madison Schwarz:** formal analysis, methodology, writing – original draft, writing – review and editing. **Matthew Hays:** data curation, formal analysis, methodology, resources, validation, visualization, writing – review and editing. **Yang Li:** data curation, formal analysis, methodology, resources, supervision, validation, visualization, writing – review and editing. **Shelley A. Johns:** conceptualization, data curation, formal analysis, funding acquisition, investigation, methodology, project administration, resources, supervision, visualization, writing – original draft, writing – review and editing.

## Ethics Statement

All participants provided informed consent. This study was approved by the Indiana University Institutional Review Board (IRB Approval Number: 11066) and was performed in accordance with US Federal Policy for the Protection of Human Subjects.

## Conflicts of Interest

The authors declare no conflicts of interest.

## Supporting information


Supporting Information S1

## Data Availability

The data that support the findings of this study are available from the corresponding author upon reasonable request.
