# Supplementary material for: Breaking the Silence: A Mixed Methods Analysis Unveiling Barriers to Mental Health Service Use in Breast Cancer Survivors With Clinically Significant Distress
Source: Psychooncology. 2026 Oct 27;35(10):e70599. doi: 10.1002/pon.70599 (PMC13714173; doi:10.1002/pon.70599)
Supplement: Supplementary file 1 [file PON-35-e70599-s001.docx]

**APPENDIX A**

**TABLE 1A** Mental health service type and respective wording on the baseline questionnaire

| Mental Health Service Type | Question on the TiC-P |
| --- | --- |
| Outpatient Mental Health Services | Have you consulted an outpatient mental health service (e.g., behavioral health consultant/specialist, community-based crisis center, community-based counselor) in the past 3 months? |
| Private Practice Services | Have you consulted a licensed psychologist, psychotherapist, or psychiatrist in a private practice setting in the past 3 months? |
| Hospital-Based Services | Have you consulted a licensed psychologist, psychotherapist, or psychiatrist in a hospital or healthcare network setting in the past 3 months? |
| Support Groups | Have you consulted a self-help or support group (such as a cancer support group, eating disorder support group, depression support group, Alcoholics Anonymous [AA], Narcotics Anonymous [NA], etc.) in the past 3 months? |

Abbreviations: TiC-P, Treatment Inventory of Costs in Patients with Psychiatric Disorders

**TABLE 2A** Outline of semi-structured interview guide with potential probing questions

| Broad Question Topic | Example Probing Questions ^†^ |
| --- | --- |
| Motivations for Study Participation | - What led you to participate in the FACing Fear study? **^‡^** - Did you perceive gaps in support for BCS that this study addressed? If so, how? - What support or insights were you hoping to gain? |
| Prior Experiences with MHS | - Have you ever used MHS (e.g., therapy, support groups)?   - If YES: Describe your experiences. Were there factors that discouraged future use (e.g., negative interactions, lack of benefit)?   - If NO: What influenced this decision (e.g., no perceived need, lack of access, stigma)? |
| Perceptions of MHS | - How would you describe your views on MHS? - What are your thoughts on individuals who attend therapy or support groups? - Would you recommend MHS to a loved one or fellow survivor? Why or why not? |
| Barriers to Accessing MHS | - What are the most significant barriers you’ve faced (e.g., cost, logistics, stigma)? - For those who considered MHS: What hesitations did you encounter? - How could healthcare systems better address these barriers (e.g., virtual options, provider outreach)? |
| Impact of Study Participation on Perceptions of MHS | - Have your views on MHS changed since enrolling in FACing Fear? - Do BCS face unique challenges in seeking mental health care? - How can psychosocial care for BCS be improved (e.g., normalizing fear of recurrence, care coordination)? |

Abbreviations: FACing Fear, **F**acilitating **A**daptive **C**op**ing** with **Fear** of Recurrence among Breast Cancer Survivors: A Three-Arm Randomized Controlled Trial; BCS, breast cancer survivors; MHS, mental health services

**^†^**For each broad topic, multiple alternative and probing follow-up questions were available to achieve a comprehensive understanding of the topics and adapt to the flow of each focus group discussion

**^‡^**“FACing Fear” was the name by which participants knew the three-arm randomized controlled trial that was the parent study for this mixed methods study

**TABLE 3A** Prevalence of self-reported clinically significant fear of cancer recurrence (FCR) at baseline as measured by the 7-Item FCR scale (FCR7)

| Clinically Significant FCR7 | Frequency | Percent |
| --- | --- | --- |
| No | 95 | 25.33 |
| Yes | **280** | **74.67** |

Note: Five subjects were removed for complete case analysis but still had clinically significant FCR7 despite missing at least one item.

**TABLE 4A** Prevalence of self-reported clinically significant distress outside of fear of cancer recurrence (as reported in Table 3A) at baseline

| Clinically Significant Distress^†^ | Frequency | Percent |
| --- | --- | --- |
| No | 156 | 40.84 |
| Yes | **226** | **59.16** |

**^†^**Distress measures included: **Anxiety Symptoms**, measured by the Generalized Anxiety Disorder scale (GAD-7); **Depressive Symptoms**, measured by the Patient Health Questionnaire (PHQ-8); **Post-Traumatic Stress Symptoms**, measured by the Impact of Events Scale-Revised (IES-R)

**TABLE 5A** Prevalence of self-reported clinically significant levels of anxiety as measured by the Generalized Anxiety Disorder scale (GAD-7)

| Clinically Significant GAD-7 | Frequency | Percent |
| --- | --- | --- |
| No | 293 | 77.72 |
| Yes | **84** | **22.28** |

Note: One subject was removed for complete case analysis but still had clinically significant GAD-7 despite missing at least one item.

**TABLE 6A** Prevalence of self-reported clinically significant levels of depressive symptoms as measured by the Patient Health Questionnaire (PHQ-8)

| Clinically Significant PHQ-8 | Frequency | Percent |
| --- | --- | --- |
| No | 286 | 76.68 |
| Yes | **87** | **23.32** |

Note: Four subjects were removed for complete case analysis but still had clinically significant PHQ-8 despite missing at least one item.

**TABLE 7A** Prevalence of self-reported clinically significant levels of post-traumatic stress symptoms as measured by the Impact of Events Scale-Revised (IES-R)

| Clinically Significant IES-R | Frequency | Percent |
| --- | --- | --- |
| No | 286 | 74.36 |
| Yes | **90** | **25.64** |

Note: 10 subjects were removed for complete case analysis but still had clinically significant IES-R despite missing at least one item.

**TABLE 8A** Comparisons of self-reported distress scores by severity category of fear of cancer recurrence (FCR) at baseline

| **Distress Measure ^†^** | **FCR Severity Category ^‡^** | **N** | **Mean** | **SD** | **Median** | **p-value** |
| --- | --- | --- | --- | --- | --- | --- |
| **GAD-7** | **Non-Clinical** | 93 | 3.17 | 3.78 | 2 | <0.0001 |
|  | **Moderate-to-Severe** | 180 | 5.21 | 4.18 | 5 |  |
|  | **Severe** | 95 | 9.72 | 5.13 | 10 |  |
| **PHQ-8** | **Non-Clinical** | 92 | 3.73 | 3.35 | 3 | <0.0001 |
|  | **Moderate-to-Severe** | 179 | 5.68 | 4.62 | 4 |  |
|  | **Severe** | 93 | 9.24 | 5.53 | 9 |  |
| **IES-R** | **Non-Clinical** | 87 | 13.78 | 8.63 | 13 | <0.0001 |
|  | **Moderate-to-Severe** | 172 | 23.44 | 12.46 | 22 |  |
|  | **Severe** | 86 | 35.48 | 13.72 | 34.5 |  |

Abbreviations: N, total sample size; SD, standard deviation

**^†^** Distress measures included: **Anxiety Symptoms**, measured by the 7-item Generalized Anxiety Disorder scale (GAD-7; total scores 0–21 with clinical cut-off ≥ 10); **Depressive Symptoms**, measured by the 8-item Patient Health Questionnaire (PHQ-8; total scores 0–24 with clinical cut-off ≥ 10); **Post-Traumatic Stress (PTS) Symptoms**, measured by the 22-item Impact of Events Scale-Revised (IES-R; total scores 0–88 with clinical cut-off ≥ 33)

**^‡^** FCR severity categories are based on the 7-item FCR scale (FCR7; total scores 6–40 with clinical cut-off ≥ 10). Following validated cut points for breast cancer survivors, baseline scores were stratified into: Non-Clinical (6–16), Clinical Moderate-to-Severe (17–26), and Clinical Severe (≥ 27).^16^

**TABLE 9A** Prevalence of self-reported clinically significant levels of any distress measure including fear of cancer recurrence at baseline

| Clinically Significant Distress^†^ | Frequency | Percent |
| --- | --- | --- |
| No | 85 | 22.19 |
| Yes | **298** | **77.81** |

**^†^**Distress measures included: **Fear of Cancer Recurrence (FCR),** measured by the 7-item FCR scale (FCR7); **Anxiety Symptoms**, measured by the Generalized Anxiety Disorder scale (GAD-7); **Depressive Symptoms**, measured by the Patient Health Questionnaire (PHQ-8); **Post-Traumatic Stress Symptoms**, measured by the Impact of Events Scale-Revised (IES-R)

**TABLE 10A** Prevalence of self-reported mental health service use within 3 months of baseline in individuals with significant distress score(s)

| Use of Any Mental Health Service^†^ | Frequency | Percent |
| --- | --- | --- |
| No | 237 | 79.53 |
| Yes | **61** | **20.47** |

**^†^**Mental health services included Outpatient Mental Health Services, Private Practice Services, Hospital-Based Services, and Support Groups

**TABLE 11A** Associations between mental health service use and clinically significant distress measures

| Mental Health Service Type^†^ | Significant Distress Measure^‡^ | Test | P-Value |
| --- | --- | --- | --- |
| Outpatient Mental Health Services | GAD-7 | Chi-Square | <.0001 |
|  | PHQ-8 | Chi-Square | <.0001 |
|  | IES-R | Chi-Square | 0.0002 |
|  | FCR7 | Chi-Square | 0.0709 |
| Private Practice Services | GAD-7 | Chi-Square | 0.0144 |
|  | PHQ-8 | Chi-Square | <.0001 |
|  | IES-R | Chi-Square | 0.0095 |
|  | FCR7 | Chi-Square | 0.6041 |
| Hospital-Based Services | GAD-7 | Fisher's Exact | 0.0043 |
|  | PHQ-8 | Fisher's Exact | 0.0036 |
|  | IES-R | Fisher's Exact | 0.0288 |
|  | FCR7 | Fisher’s Exact | 0.3775 |
| Support Groups | GAD-7 | Chi-Square | 0.0794 |
|  | PHQ-8 | Chi-Square | 0.2717 |
|  | IES-R | Chi-Square | 0.9306 |
|  | FCR7 | Chi-Square | 0.7113 |
| Any Mental Health Service | GAD-7 | Chi-Square | 0.0030 |
|  | PHQ-8 | Chi-Square | 0.0015 |
|  | IES-R | Chi-Square | 0.0387 |
|  | FCR7 | Chi-Square | 0.9423 |

**^†^**Mental health services included Outpatient Mental Health Services, Private Practice Services, Hospital-Based Services, and Support Groups;

**^‡^**Distress measures included: **Fear of Cancer Recurrence (FCR),** measured by the 7-item FCR scale (FCR7); **Anxiety Symptoms**, measured by the Generalized Anxiety Disorder scale (GAD-7); **Depressive Symptoms**, measured by the Patient Health Questionnaire (PHQ-8); **Post-Traumatic Stress Symptoms**, measured by the Impact of Events Scale-Revised (IES-R)

**TABLE 12A** Associations between high score yield on distress measure and frequency of mental health service use

| Distress Measure^†^ | Log-Count of Mental Health Service Visits by Type | Poisson Regression Estimate (PRE) ^‡^ | *P*-Value | Exponentiated PRE^‡^ |
| --- | --- | --- | --- | --- |
| FCR7 | Outpatient Mental Health Services | 0.0324 | 0.0117 | 1.03 |
| GAD-7 | Outpatient Mental Health Services | 0.1281 | <.0001 | 1.14 |
| PHQ-8 | Outpatient Mental Health Services | 0.1304 | <.0001 | 1.14 |
| IES-R | Outpatient Mental Health Services | 0.0494 | <.0001 | 1.05 |
| FCR7 | Private-Practice Services | -0.0209 | 0.0931 |  |
| GAD-7 | Private-Practice Services | 0.0918 | <.0001 | 1.10 |
| PHQ-8 | Private-Practice Services | 0.1577 | <.0001 | 1.17 |
| IES-R | Private-Practice Services | 0.0013 | 0.8345 |  |
| FCR7 | Hospital-Based Services | -0.0137 | 0.4589 |  |
| GAD-7 | Hospital-Based Services | 0.1107 | <.0001 | 1.12 |
| PHQ-8 | Hospital-Based Services | 0.1005 | <.0001 | 1.11 |
| IES-R | Hospital-Based Services | 0.0384 | <.0001 | 1.04 |
| FCR7 | Support Groups | -0.0472 | <.0001 | 0.95 |
| GAD-7 | Support Groups | 0.0927 | <.0001 | 1.10 |
| PHQ-8 | Support Groups | 0.0726 | <.0001 | 1.08 |
| IES-R | Support Groups | -0.0079 | 0.0623 |  |
| FCR7 | Any Mental Health Service | 0.0230 | 0.1134 |  |
| GAD-7 | Any Mental Health Service | 0.0967 | <.0001 | 1.10 |
| PHQ-8 | Any Mental Health Service | 0.1050 | <.0001 | 1.11 |
| IES-R | Any Mental Health Service | -0.0338 | <.0001 | 1.03 |

**^†^**Distress measures included: **Fear of Cancer Recurrence (FCR),** measured by the 7-item FCR scale (FCR7); **Anxiety Symptoms**, measured by the Generalized Anxiety Disorder Scale (GAD-7); **Depressive Symptoms**, measured by the Patient Health Questionnaire (PHQ-8); **Post-Traumatic Stress Symptoms**, measured by the Impact of Events Scale-Revised (IES-R).

**^‡^**PRE represents the average increase in log-count of MHS visits per unit increase of the distress measure. When significant, the exponentiated PRE further shows the average factor by which MHS visits’ count changed per unit increase of the distress measure.

**FIGURE 1A** Relationship between fear of cancer recurrence and cancer-related avoidant coping (*ρ* = .643, *p* < .001)
